# Intraocular Foreign Bodies: Clinical Characteristics and Prognostic Factors Influencing Visual Outcome and Globe Survival in 373 Eyes

**DOI:** 10.1155/2019/5208092

**Published:** 2019-02-13

**Authors:** Yang Liu, Shuang Wang, Ying Li, Qiaoyun Gong, Guanfang Su, Jinsong Zhao

**Affiliations:** Eye Center, The Second Hospital of Jilin University, 218 Ziqiang Street, Changchun, Jilin 130021, China

## Abstract

**Aim:**

To describe epidemiologic and clinical characteristics and prognostic factors influencing visual outcome after intraocular foreign bodies (IOFBs) injury.

**Methods:**

Medical records of 370 patients (373 eyes) with IOFBs were reviewed to identify the factors influencing visual acuity by univariate and multivariate analyses.

**Results:**

The majority of patients (97.0%) were men, with a mean age of 38.1 years. The most common cause of ocular injury was hammering (52.6%); magnetic IOFBs occurred in 84.7% of these cases. Factors associated with poor visual outcome (defined as <1.3 logMAR) included the following: age ≥50 years (*P*=0.046); worse presenting visual acuity (*P* < 0.001); complications of retinal breaks (*P*=0.006) and endophthalmitis (*P*=0.032); vitrectomy (*P*=0.035); and intraocular C_3_F_8_ gas tamponade (*P*=0.038). Excellent visual outcome (defined as ≥0.5 logMAR) was associated with age <50 years (*P*=0.003); better presenting visual acuity (PVA) (*P* < 0.001); wound length <4 mm (*P*=0.005); absence of vitreous hemorrhage (*P*=0.026) and retinal breaks (*P* < 0.001); nonvitrectomy surgery (*P*=0.043); and use of balanced saline (*P*=0.029).

**Conclusions:**

Multiple prognostic factors were identified that may predict visual outcome and globe survival after IOFBs injury. Age, initial presenting visual acuity, wound length, complications (vitreous hemorrhage, retinal breaks, and endophthalmitis), surgical approach, and intraocular tamponade were significant predictors of visual outcome.

## 1. Introduction

Intraocular foreign bodies (IOFBs) are a leading cause of visual morbidity and blindness, especially in the working population [1, 2]. The management of IOFBs is a major challenge to the ophthalmologist, due to their specific clinical implications, as well as the diversity of associated changes, the severity of complications, and the specificity of diagnosis and treatment. Prognosis for vision in IOFBs was dependent on confluent predictive factors, including age, length of wound, time between injury and repair, volume of IOFBs, and complications such as relative afferent pupillary defect (RAPD), retinal detachment and endophthalmitis, as published by previous authors [3, 4]. However, most of the available data derived from other world populations outside China. As such, more comprehensive updated data are required. In this study, we evaluated 373 eyes with IOFBs; here, we present our findings, based on the current literatures, to determine the potential prognostic factors and analyze the efficiency of the surgical procedures.

## 2. Subjects and Methods

All medical and surgical records of patients admitted between 2009 and 2014 were retrospectively reviewed on condition of anonymity. Written informed consent for inclusion in research procedures was obtained from all patients during the course of clinical practice. The plan was approved by the Ethics Committee of the Second Hospital of Jilin University. Demographic data included age, sex, length of wound, entry site, and time between injury and repair. We defined characteristic IOFBs as metal with the help of helical CT performed for determining the type of IOFBs. CT was accurate at detecting and localizing intraocular metallic, glass, and stone foreign bodies. It showed wood was the least dense of the nonmetallic foreign bodies, followed by plastic and then glass. Metal fragments were hyperdense and caused artifacts. IOFBs can cause direct mechanical damage along their path of entry into the eye. CT and magnetic resonance imaging (MRI) have emerged as important imaging techniques of choice for the evaluation of not only IOFBs but also injured eyes. The principal advantage of MRI is that it can safely and accurately detect wood and plastic IOFBs. Other variables including initial and final best-corrected visual acuities (BCVA); detailed information about IOFBs, such as the size, number, type, and location; and the time before IOFBs removal were obtained from the patients' records. Complications such as cataract, vitreous hemorrhage, retinal breaks (breaks in the retina can be categorized as a tear or a hole), retinal detachment, and endophthalmitis were also noted along with the associated diagnostic studies and procedures. The study did not include RAPD as a predictive factor because of the limited number of cases. Therefore, it could not be determined as a probable prognostic factor for poor visual outcomes because of statistical bias. Eyes with posttraumatic endophthalmitis, rather than postoperative endophthalmitis, were included in this study. In particular, all eyes with retained IOFBs and associated cataract were investigated and analyzed in detail. Patients with a prior history of ocular disease, previous ocular trauma, and/or incomplete data were excluded.

Following a thorough preoperative workup, operative considerations included the timing of surgery (delayed versus immediate), feasibility of primary cataract extraction and IOL implantation, and instruments and route used for IOFBs removal. All corneal wounds were sutured with 10/0 Ethilon, whereas scleral wounds were sutured with 6/0 Vicryl or 5/0 PremiCron. The timing of cataract extraction was not consistent and depended on the presence of endophthalmitis, properties of IOFBs, and lens status (opacification, capture, or dislocation). Accordingly, emergency surgery or scheduled surgery was performed. In select cases, primary IOL placement was not well tolerated and may increase the risk of endophthalmitis or other complications. Secondary IOL implantation was more easily performed at 3 months after cataract extraction. Scheduled surgery helped clearing of fibrin and intraocular inflammation and allowed proper determination of the lens position, capsular integrity, and IOL power. Several factors, including the presence or absence of clinical endophthalmitis, tolerability of the patient for an extended surgical procedure, and availability of well-trained operating room personnel were considered carefully when we decided to perform IOFBs removal at the time of primary globe repair or delayed IOFBs removal.

Removal of anterior chamber IOFBs through the entry wound is generally not recommended. The foreign body was removed using an intraocular magnet or forceps through a secondary corneal limbal incision, which can also be used for the removal of intralenticular foreign bodies using a forceps or magnet. We usually made a decision according to the necessity of combined iris root incision or cataract surgery. Scleral incision was usually used for ciliary foreign bodies, if necessary, combined with iris root incision when IOFBs were located in the ciliary body crown. External magnet was used for cases with feasibility of immediate extraction and good fundus visualization with minimal associated posterior segment injury. For small and some medium-sized objects located in the ciliary body and vitreous or embedded in the entry wound, as well as IOFBs in the eyes with endophthalmitis showing contraindications for vitrectomy, magnet extraction was performed if the magnetic test showed a positive result. The purpose of this test was to determine whether the foreign body in the eye was magnetic and the possibility of extracting the IOFBs with an external magnet by placing the magnet at the site of pars plana which was closest to the foreign bodies' location. The test result was noted as positive when bouncing of sclera and/or IOFBs movement was observed. The external magnet was used to remove intravitreal IOFBs or relatively free unimpacted IOFBs. During the process of external magnet, we avoided moving the magnet towards the sclera repeatedly as the IOFBs may move erratically and damage the ocular structures. Then, a 4 mm sclerotomy starting from the limbus was performed. When the total volume of the foreign body indicated a medium to large size, T-shaped sclerotomy was performed for IOFBs removal. Smaller foreign bodies can be removed at the sclerotomy site. Alternatively, it is helpful to preplace a mattress suture so that an enlarged sclerotomy incision can be quickly closed to decrease the period of hypotony after IOFBs removal.

When a patient was unstable for an extended surgical procedure, vitrectomy and/or lensectomy were performed. Vitrectomy was performed by two senior specialists using the three-incision 20G technique, with complete vitreous removal. IOFBs could be removed with maximum control using IOFBs forceps or an intraocular magnet inserted through the pars plana sclerotomy site (<5 mm). The use of forceps or a magnet depended on the nature of IOFBs (magnetic or nonmagnetic). The IOFBs forceps was also used to grasp the foreign body and bring IOFBs into the anterior chamber (>5 mm), followed by IOFBs extraction through the limbus incision. The operation was performed under the condition of crystalline lens removal with the vitrectomy cutter or with the ultrasonic fragmatome. It was important to make a limbus incision or a sclerotomy that was large enough for passage of the foreign body though the globe without incarceration during vitrectomy. After IOFBs removal, a complete retinal examination was performed under scleral depression in order to check for retinal tears, retinal detachment, and/or choroidal detachment. Retinal tear or hole was treated with laser photocoagulation or retinal cryopexy. When retinal detachment was present, vitrectomy with gas or silicone oil endotamponade was considered. The material for endotamponade was selected as the authors' personal surgical experiences and preferences and the severity of the retinopathy. The indications for silicone oil endotamponade have been extended to giant retinal tears, retinal detachments, complicated pediatric retinal detachments, and endophthalmitis. The most common indications for intraocular gas injection include retinal detachment surgery with vitrectomy, pneumatic retinopexy, displacement of subretinal hemorrhage, and postvitrectomy gas exchange in vitrectomized eyes.

BCVA values were converted to logarithm of the minimum angle of resolution (logMAR) units for the purpose of analysis. According to the 2003 World Health Organization classification of vision loss and blindness [5], a final BCVA of 0.5 logMAR or better was defined as excellent recovery of visual acuity, while a final BCVA of 1.3 logMAR was considered a poor recovery. Larger logMAR value means worse visual acuity. For example, logMAR values of 2, 3, 3.5, and 4 were assigned to visual acuities of count fingers at 1 foot (CF 1), hand motion (HM), light perception (LP), and no light perception (NLP), respectively, according to a previous report [6].

All data were collected, reviewed for errors, and entered into an electronic database. Univariate and multivariate statistical analyses were performed using Pearson's chi-square tests and logistic regression. A *P* value of <0.05 was considered statistically significant.

## 3. Results

### 3.1. General Information

The study population included 370 patients (373 eyes) with an average follow-up period of 10.2 months (range: 4.3–48 months) after surgical intervention. The patient age ranged from 5 to 71 years (mean age, 38.1 years). Most of the patients were men (97.0%), the majority of whom were employed in industrial and agricultural production units. Tool-related activity, particularly hammering (52.6%), was the most common cause of IOFBs injuries. The material of the foreign bodies varied as follows: magnetic, 84.72%; nonmagnetic metal, 2.14%; glass, 4.02%; plastic, 0.80%; stone, 5.63%; vegetable, 1.60%; and eyelash, 1.07%. Failed extraction was observed for 22 of the 159 eyes treated with external magnet. Further analysis of our data revealed that one, one, and two eyes in the external approach group developed retinal detachment, endophthalmitis, and lens opacity, respectively. Complications of vitrectomy (144 eyes) included silicone oil in the anterior chamber in two eyes, cataract formation in six eyes, low intraocular pressure in two eyes, and hemorrhage in two eyes. Retinal detachment (7 eyes) and PVR (9 eyes) were the predominant complications leading to multiple surgeries in the vitrectomy group.

### 3.2. Visual Acuity before and after Surgery

We first analyzed the trends of preoperative and postoperative examinations of visual acuity. As listed in Table 1, among these patients, 24.9% of those injuries caused by IOFBs presented with a presenting visual acuity (PVA) better than 0.5 logMAR, while this proportion was significantly increased to 36.7% after surgery (*P* < 0.001). Between preoperatively and postoperatively among the medium group, there was no significant difference (*P*=0.07). Among the poor visual acuity group, 57.4% had PVA worse than 1.3 logMAR, while this proportion was significantly reduced to 40.0% (*P* < 0.001). In general, the overall logMAR of BCVA improved significantly after IOFBs removal (*P* < 0.001, chi-square test) (Table 1).

### 3.3. Treatment and Effects of Traumatic Cataract

The most common ocular complication related to IOFBs was traumatic cataract. Ninety-one patients presented with traumatic cataract and underwent IOL implantation. Surgical procedures included the following: (1) combined IOFBs removal, cataract extraction, and simultaneous IOL implantation (single-stage procedure); (2) IOFBs removal combined with cataract extraction and subsequent IOL implantation or IOFBs removal with subsequent cataract extraction and IOL implantation (two-stage procedure); and (3) IOFBs removal, cataract extraction, and IOL implantation (three-stage procedure). In both the BCVA <1.3 logMAR and BCVA ≥0.5 logMAR groups, there was no effect on visual outcomes when the procedures were combined (*P*=0.125) or separate (*P*=0.183). In general, surgical therapy improved visual outcome for lens injury and was not predictive of poor vision (Table 2).

### 3.4. Predictive Factors for the Visual Outcome According to Univariate Analysis

For detection of the predictive factors for the visual outcome, patients were divided into two groups according to the final BCVA: excellent visual outcome (final BCVA, ≥0.5 logMAR) and poor visual outcome (final BCVA, <1.3 logMAR). Univariate statistical analysis was performed to identify prognostic variables associated with each of these two groups (Table 3). The results revealed that a poor final BCVA was associated with the following factors: age, ≥50 years; PVA, <0.5 logMAR; length of the entry wound, ≥10 mm; interval before wound repair, ≥24 h; presence of large IOFBs; presence of nonmagnetic IOFBs; IOFBs located in the ciliary body and retina; and complications developed. Vitrectomy, multiple surgeries, and gas tamponade were also significant negative predictors.

On the contrary, an excellent visual outcome was significantly associated with a younger age, better PVA, wounds smaller than 4 mm, self-sealing wounds, and small IOFBs, single and magnetic foreign bodies, intralenticular foreign bodies, and lack of complications. The use of an external magnet, nonvitrectomy surgery, and a single surgery were also significant predictors of an excellent visual outcome.

### 3.5. Predictive Factors for the Visual Outcome According to Multivariate Analysis

For further clarification of independent risk factors for a poor visual outcome, multivariate statistical analysis was performed (Table 4), and it revealed that an age of ≥50 years (*P*=0.046), a PVA of <0.5 logMAR (*P* < 0.001), retinal breaks (*P*=0.006), and endophthalmitis (*P*=0.032) were significant predictors of a poor visual outcome. Moreover, vitrectomy (*P*=0.035) and intraocular C_3_F_8_ tamponade (*P*=0.038) were independent risk factors for a final BCVA of <1.3 logMAR.

On the contrary, independent factors for an excellent visual outcome were as follows: age <50 years (*P*=0.003); PVA ≥0.5 logMAR (*P* < 0.001); wound length <4 mm (*P*=0.005); absence of vitreous hemorrhage (*P*=0.026) and retinal breaks (*P* < 0.001); nonvitrectomy surgery (*P*=0.043); and use of balanced salt solution during surgery (*P*=0.029) (Table 5). When compared with cases where gas or oil tamponade was used, cases that did not require postoperative tamponade showed better visual outcomes.

## 4. Discussion

IOFBs could result in severe tissue disruption and visual loss. The population at greatest risk for IOFBs is young male industrial workers. IOFBs occurrence in this population has been consistent over time. Previous reports investigating the characteristics and prognostic factors of IOFBs were largely retrospective [1, 7–9]. Our study suffers from the same limitation. However, we further elucidated the risk factors affecting visual outcome and complications. The aim of our study was to evaluate the changes in BCVA, determine prognostic factors, assess ocular complications, and analyze the efficiency of surgical procedures for IOFBs injury.

Our results showed lack of improvement of BCVA in patients ≥50 years of age, consistent with a previous multivariate analysis [10]. Older persons are at high risk for endophthalmitis developing after retaining an IOFB [11]. In our study, patients over 50 years had a significantly higher probability of endophthalmitis than the other groups identified by univariate analysis (data not shown, *P*=0.022). Consistent with other clinical studies, we found that PVA was an important prognostic indicator of visual outcome after IOFBs trauma [12–14]. Similar to our study, Williams et al. did not find a predictive value for the wound location, but a wound of ≥4 mm had an adverse effect on the visual outcome [15]. An open wound caused by a perforating eye injury should thus be repaired as soon as possible. Self-sealing of eye wounds protects against the prolapse of tissue and the development of endophthalmitis; consistent with this observation, self-sealing is a significant predictive factor of a positive outcome.

Several authors stress that the most important predictive factor for prognosis is the size of the IOFBs [16, 17]. Increasing IOFBs size is associated with an increase in the frequency of uveal prolapse, intraocular cataract, hemorrhage, and worsened final visual acuity. Unlike the study by Thompson et al. [11], we found a significant correlation between nonmagnetic IOFBs material and worse visual outcomes. In our study, the location of the IOFBs was associated with poor visual results, in agreement with previous observations [18]. We found that an IOFB located at the ciliary body and retina results in more mechanical damage than an IOFB in the anterior chamber, lens, or vitreous. Controversies in the management of IOFBs eye injuries focus on whether the IOFBs should be immediately removed. Recent studies suggest that delay in the removal of an IOFB may not be as critical to vision preservation as previously thought [19]. Similar to our study, Wickham et al. found no significant association between time before removal of IOFBs and poor visual outcomes. Immediate removal of the IOFBs has been reported to reduce the risk of endophthalmitis [20]. However, delayed surgical intervention is preferred in young patients, since complete removal of the posterior hyaloid is difficult, and there is increased risk of intraoperative hemorrhage in an inflamed eye. Removal of the IOFBs at an average of 31.9 days after injury is longer than reported in other studies; however, this delay did not influence the visual outcome [18]. Other studies have not found the interval before surgery to be a significant factor for visual outcome [21, 22].

Some traumatic complications influence eventual visual acuity. These include vitreous hemorrhage, retinal breaks and retinal detachment, proliferative vitreoretinopathy (PVR), and endophthalmitis. Traumatic cataract had no effect on the final visual acuity, this results the same as with by the present investigators [23]. Vitreal haemorrhages were not the significant factors for worse vision [17, 24] because it can be successfully cured by surgery. In our study, damage or loss of retinal photoreceptor cells from retinal detachment resulted in irreversible vision damage, especially involving the macula. The presence of initial or subsequent retinal detachment is a significant predictor of poor visual outcome, as reviewed previously [25, 26]. Cardillo et al. found that the relative risk for an unfavorable outcome was 11.7 times greater in eyes with PVR, compared with eyes that did not develop PVR [27]. PVR is a serious complication that is difficult to manage with currently available treatment methods. Endophthalmitis is a serious complication, with important therapeutic and prognostic implications [17, 28]. Endophthalmitis can cause severe tissue damage, resulting in the need for enucleation. Poor visual outcome is significantly associated with endophthalmitis.

Primary management of IOFBs with vitrectomy or magnet extraction is controversial due to the different characteristics and advantages. In our experience, surgeons prefer the use of electromagnetic extraction for posterior segment IOFBs, regardless of the presence of minimal or moderate vitreous hemorrhage. Vitrectomy techniques provide more precise localization and visualization for the extraction of IOFBs. However, primary extraction with vitrectomy in an emergency can be difficult, and iatrogenic retinal injuries can occur due to the absence of posterior vitreous detachment, risk of bleeding, and accidental retinal tear. Chow et al. stated that in selected cases, the external approach for removal of magnetic IOFBs is an acceptable option in improving the visual outcome [29]. This finding differs from the previous work which showed no significant differences in the visual outcome when internal and external approaches were compared [30]. In our study, additional operations were required in 55.2% of the eyes. Additional surgery may be necessary to reconstruct the eyeball and improve the visual outcome; however, the requirement for repeat surgery is associated with a poorer visual outcome [15, 29].

Another factor influencing the results of our study was the use of silicone oil or intraocular gases for intraocular tamponade. Although the use of silicone oil has potential risks, including secondary glaucoma and bullous keratopathy, the use of intraocular gases presents a higher risk for worse visual outcomes. When compared with intraocular gas, silicone oil tamponade was more effective in securing retinal reattachment and confining intraocular inflammation [23].

## 5. Conclusion

This review reflects the general status of eyes with IOFBs in our area, from 2009 through 2014. We found that patient age ≥ 50 years, poor initial presenting VA, retinal breaks, endophthalmitis, surgical vitrectomy, and intraocular tamponade were independent risk factors, as assessed by multivariate analysis. Our prognostic factors for visual outcome of an IOFBs injury might be useful to evaluate the severity of trauma, determine the appropriate therapy, and more accurately predict prognosis. In addition, because our follow-up period is not long enough though the principal condition of ocular remains stabilized, some problems still need to be addressed. We hope that our experience can help oculists to improve their understanding of IOFBs injury.

## Figures and Tables

**Table 1:** Visual acuity in 373 eyes before and after removal of intraocular foreign bodies.

Visual acuity (logMAR)	PVA no. (%)	Postoperative BCVA no. (%)	*P* value
Good: 0.5 or better	93 (24.9%)	137 (36.7%)	<0.001
Medium: 1.3 to 0.5	66 (17.7%)	87 (23.3%)	0.07
Poor: 1.3 or worse	214 (57.4%)	149 (40.0%)	<0.001

PVA: presenting visual acuity; BCVA: best-corrected visual acuity.

**Table 2:** Surgical procedures and visual outcome in patients of traumatic cataract with IOFBs.

	No. (%)	BCVA <1.3 logMAR	BCVA ≥0.5 logMAR
		No. (%)	No. (%)
IOFBs removal simultaneous CE + IOL implantation	29 (31.9)	6 (20.7)	15 (51.7)
IOFBs removal simultaneous CE + secondary IOL implantation	32 (35.1)	4 (12.5)	16 (50.0)
IOFBs removal subsequent CE + IOL implantation	13 (14.3)	4 (30.8)	4 (30.8)
IOFBs removal subsequent CE + secondary IOL implantation	17 (18.7)	7 (41.2)	4 (23.5)
*P* value		0.125	0.183

**Table 3:** Univariate analysis on features of eyes with IOFBs affecting BCVA (BCVA <1.3 logMAR and BCVA ≥0.5 logMAR).

Variables	BCVA		*P* value	BCVA		*P* value
	<1.3 logMAR no. (%)	≥1.3 logMAR no. (%)		<0.5 logMAR no. (%)	≥0.5 logMAR no. (%)	
Age (years)						
<18	7 (35.0)	13 (65.0)	0.020	8 (40.0)	12 (60.0)	0.002
18∼50	101 (36.5)	176 (63.5)		169 (61.0)	108 (39.0)	
≥50	41 (53.9)	35 (46.1)		59 (77.6)	17 (22.4)	

Presenting VA						
<0.5 logMAR	140 (50.0)	140 (50.0)	<0.001	221 (78.9)	59 (21.1)	<0.001
≥0.5 logMAR	9 (9.7)	84 (90.3)		15 (16.1)	78 (83.9)	

Length of wound						
0∼4 mm	77 (32.4)	161 (67.6)	<0.001	126 (52.9)	112 (47.1)	<0.001
4∼10 mm	52 (49.5)	53 (50.5)		85 (81.0)	20 (19.0)	
10 mm or longer	20 (66.7)	10 (33.3)		25 (83.3)	5 (16.7)	

Entry site of IOFBs						
I zone	108 (38.8)	170 (61.2)	0.395	172 (61.9)	106 (38.1)	0.612
II zone	31 (47.0)	35 (53.0)		45 (68.2)	21 (31.8)	
III zone	10 (34.5)	19 (65.5)		19 (65.5)	10 (34.5)	

Time before primary repair						
Self-sealing	37 (27.6)	97 (72.4)	0.001	68 (50.7)	66 (49.3)	<0.001
<24 h	95 (46.1)	111 (53.9)		141 (68.4)	65 (31.6)	
≥24 h	17 (51.5)	16 (48.5)		27 (81.8)	6 (18.2)	

Surface of IOFBs						
Small: <4 mm^2^	15 (30.0)	35 (70.0)	<0.001	24 (48.0)	26 (52.0)	<0.001
Medium: 4∼16 mm^2^	103 (36.3)	181 (63.7)		176 (62.0)	108 (38.0)	
Large: >16 mm^2^	31 (79.5)	8 (20.5)		36 (92.3)	3 (7.7)	

Multiple IOFBs						
Yes	15 (57.7)	11 (42.3)	0.055	19 (73.1)	7 (26.9)	0.282
No	134 (38.6)	213 (61.4)		217 (62.5)	130 (37.5)	

Magnetic of IOFBs						
Yes	118 (37.3)	198 (62.7)	0.016	193 (61.1)	123 (38.9)	0.038
No	31 (54.4)	26 (45.6)		43 (75.4)	14 (24.6)	

Location of IOFBs						
Anterior chamber	12 (25.0)	36 (75.0)	0.001	22 (45.8)	26 (54.2)	<0.001
Intralens	6 (22.2)	21 (77.8)		9 (33.3)	18 (66.7)	
Ciliary body	12 (66.7)	6 (33.3)		15 (83.3)	3 (16.7)	
Vitreous	59 (36.9)	101 (63.1)		102 (63.7)	58 (36.3)	
Retina	60 (50.0)	60 (50.0)		88 (73.3)	32 (26.7)	

Time before IOFBs removal						
Within 24 h	77 (38.3)	124 (61.7)	0.675	122 (60.7)	79 (39.3)	0.427
24∼72 h	30 (43.5)	39 (56.5)		51 (73.9)	18 (26.1)	
72 h∼1 week	20 (47.6)	22 (52.4)		25 (59.5)	17 (40.5)	
1∼2 weeks	6 (28.6)	15 (71.4)		14 (66.7)	7 (33.3)	
2 weeks∼1 month	9 (45.0)	11 (55.0)		13 (65.0)	7 (35.0)	
3 months or longer	7 (35.0)	13 (65.0)		11 (55.0)	9 (45.0)	

Cataract						
Yes	128 (42.0)	177 (58.0)	0.091	205 (67.2)	100 (32.8)	0.001
No	21 (30.9)	47 (69.1)		31 (45.6)	37 (54.4)	

Vitreous hemorrhage						
Yes	90 (51.7)	84 (48.3)	<0.001	138 (79.3)	36 (20.7)	<0.001
No	59 (29.6)	140 (70.4)		98 (49.2)	101 (50.8)	

Retinal breaks					
Yes	70 (57.9)	51 (42.1)	<0.001	100 (82.6)	21 (17.4)	<0.001
No	79 (31.3)	173 (68.7)		136 (54.0)	116 (46.0)	

Retinal detachment						
Yes	42 (59.2)	29 (40.8)	<0.001	58 (81.7)	13 (18.3)	0.001
No	107 (35.4)	195 (64.6)		178 (58.9)	124 (41.1)	

PVR						
Yes	34 (52.3)	31 (47.7)	0.025	58 (89.2)	7 (10.8)	<0.001
No	115 (37.3)	193 (62.7)		178 (57.8)	130 (42.2)	
Endophthalmitis					
Yes	34 (61.8)	21 (38.2)	<0.001	47 (85.5)	8 (14.5)	0.001
No	115 (36.2)	203 (63.8)		189 (59.4)	129 (40.6)	

Surgery approaches						
Magnet	50 (31.4)	109 (68.6)	0.001	87 (54.7)	72 (45.3)	0.002
Vitrectomy	74 (51.4)	70 (48.6)		107 (74.3)	37 (25.7)	
Forceps or phaco	25 (35.7)	45 (64.3)		42 (60.0)	28 (40.0)	

Multiple surgeries						
Yes	95 (46.1)	111 (53.9)	0.007	153 (74.3)	53 (25.7)	<0.001
No	54 (32.3)	113 (67.7)		83 (49.7)	84 (50.3)	

Tamponade used						
Nonvitrectomy	74 (32.3)	155 (67.7)	<0.001	128 (55.9)	101 (44.1)	<0.001
Gas implantation	29 (70.7)	12 (29.3)		33 (80.5)	8 (19.5)	
Silicone injection	28 (50.9)	27 (49.1)		46 (83.6)	9 (16.4)	
Balanced saline	18 (37.5)	30 (62.5)		29 (60.4)	19 (39.6)	

**Table 4:** Multivariate analysis of independent risk factors for BCVA (BCVA <1.3 logMAR) in eyes with IOFBs.

Variables	*P*	OR	95% CI
Age (≥50 years)	0.046	0.619	0.373 to 1.028
Worse presenting VA	<0.001	8.625	3.819 to 19.482
Retinal breaks	0.006	0.380	0.190 to 0.761
Endophthalmitis	0.032	0.460	0.226 to 0.935
Surgery approaches	0.035	0.674	0.467 to 0.973
Tamponade used	0.038	1.364	1.017 to 1.830

OR: odds ratio; 95% CI: 95% confidence interval.

**Table 5:** Multivariate analysis of independent risk factors for BCVA (BCVA ≥0.5 logMAR) in eyes with IOFBs.

Variables	*P*	OR	95% CI
Age (<50 years)	0.003	0.373	0.197 to 0.707
Better presenting VA	<0.001	27.206	11.351 to 65.203
Length of wound (<4 mm)	0.005	0.450	0.258 to 0.786
Vitreous hemorrhage	0.026	0.445	0.218 to 0.907
Retinal breaks	<0.001	0.165	0.060 to 0.455
Surgery approaches	0.043	0.680	0.435 to 1.064
Tamponade used	0.029	1.447	1.039 to 2.016

OR: odds ratio; 95% CI: 95% confidence interval.

## Data Availability

The data used to support the findings of this study are available from the corresponding author upon request.
